# The Triple “S” Impact of COVID-19: Nationwide Evidence of the Impact of the Stress Associated With Restrictive Measures on Substance Use, Sleep, and Social Connectedness in Qatar

**DOI:** 10.1097/PRA.0000000000000737

**Published:** 2023-09-07

**Authors:** Muna Abed Alah, Sami Abdeen, Vahe Kehyayan, Iheb Bougmiza

**Affiliations:** ABED ALAH and ABDEEN: Community Medicine Department, Hamad Medical Corporation (HMC), Doha, Qatar; KEHYAYAN: Associate Professor, Healthcare Administration, College of Business Management, University of Doha for Science & Technology, Doha, Qatar; BOUGMIZA: Consultant, Community Medicine Department, Primary Health Care Corporation (PHCC), Doha, Qatar, and Associate Professor, Community Medicine Department, College of Medicine, Sousse University, Tunisia.

**Keywords:** COVID-19, restrictive measures, substance use, sleep, social connectedness, Qatar

## Abstract

**Objectives:**

Countries worldwide implemented social and movement restrictions to contain the spread of coronavirus disease 2019 (COVID-19). Unfortunately, such restrictions have adversely impacted people’s lifestyles. The goal of this study was to assess the impact of COVID-19-related restrictive measures on substance use, sleep, and social connectedness in Qatar’s population.

**Methods:**

A web-based survey was conducted between January 2021 and February 2021 targeting adults ≥18 years of age who were residing in Qatar between March and August 2020.

**Results:**

A total of 1408 participants completed the survey. Of tobacco users in our sample, 36% reported increased tobacco use since the start of home confinement, while 41.6% of alcohol users reported decreased alcohol use. Concerning sleep, 46.1% reported an increase in average sleep duration per day (0.77 h mean increase, 95% CI: 0.66-0.88, *P*<0.001), and a third of participants reported poorer sleep quality. Of the participants, 39.6% felt socially disconnected. Perceived stress was found to be an independent predictor for increased use of tobacco, deterioration in sleep quality, and increased sleep duration during home confinement.

**Conclusions:**

Restrictive measures related to COVID-19 resulted in both positive and negative impacts on the lifestyle of Qatar’s population. Emphasis should be placed on encouraging people to adopt healthy strategies for coping with various stressors that arise during future home confinement measures. It is also necessary to address the persistence of adverse consequences in the postpandemic era.

The coronavirus disease 2019 (COVID-19) pandemic forced governments in various countries to take strict protective measures as a step to mitigate the spread of the infection. Such measures included imposing travel restrictions, closing shopping malls, businesses, schools, and universities, and canceling public events. Some countries adopted even more strict measures such as imposing total lockdowns or even curfews.^1,2^ Unfortunately, with such mandated restrictions, some people experienced significant distress in the form of anxiety, anger, and other symptoms of stress.^3,4^ Recent evidence has suggested that individuals who are kept in isolation and quarantine during infectious outbreaks experience several stressors, such as fear about their own health or fear of infecting others,^5–7^ boredom, frustration, and a sense of isolation from the rest of the world resulting from confinement, loss of usual routine, and reduced social and physical contact with others.^5,6,8,9^

The stress surrounding the COVID-19 pandemic led to sleep disturbances^10^ which in turn worsened stress levels, giving rise to a dangerous vicious cycle.^11^ Stress and sleep disturbances can stimulate tobacco cravings and decrease the ability to resist smoking and potentiate smoking intensity.^12,13^ They can also result in increased alcohol consumption.^14^ In March 2020, Qatar implemented several preventive measures to contain the spread of COVID-19, such as limiting the number of people going to work and switching them to working from home, closure of educational institutions, shopping malls, gyms, and restaurants, discouraging social gatherings, and strongly emphasizing the importance of home confinement through various media channels.

Easing and lifting of restrictions occurred in four distinct phases.^15^ Commencing on June 15, 2020, the first phase marked the beginning of this gradual process, which culminated in the fourth phase commencing on September 15, 2020. Regrettably, Qatar encountered delays and was unable to attain phase 4 within the intended timeframe to fully resume normal activities.

To provide further context, this study was conducted between January 2021 and February 2021, during which the majority of restrictions had been relaxed. However, despite these efforts, the nation had not achieved a “normal state” with the complete restoration of operational services as initially planned. In fact, many people continued working from home; restaurants, gyms, and malls were open with very limited capacities; and the government continued emphasizing and encouraging the importance of staying at home and strongly discouraged social gatherings.

This study is part of a bigger national study that assessed the impact of COVID-19 on several aspects of the lifestyle of Qatar’s population. The first part was published previously and assessed the impact on diet, physical activity, and body weight.^16^ In this study, our goal was to assess the impact of the stress of home confinement during the COVID-19 pandemic on other aspects of lifestyle we referred to as triple “S,” which includes substance use particularly smoking and alcohol use, sleep, and social connectedness among the public in Qatar. To our knowledge, studies exploring these aspects of lifestyle during home confinement or lockdown measures in the Middle East are limited. Our objectives were to explore changes in tobacco and alcohol use, sleep duration and quality, and social connectedness and their associated factors. We believe that evidence derived from this study will guide the implementation of effective lifestyle-related interventions targeting such areas both currently and during any potential future pandemics that mandate periods of movement restriction and home confinement.

## METHODS

### Study Design and Target Population

A national cross-sectional survey was conducted between January 2021 and February 2021. The target population included adults ≥18 years of age who stayed in Qatar for at least 2 months between March and August 2020, which is the time when the State of Qatar announced strict public health measures and emphasized the importance of home confinement.

### Study Procedure

We developed a web-based, self-administered survey using SurveyMonkey software. The link to the survey was posted on the social media platforms of Hamad Medical Corporation (eg, Instagram, Facebook, and Twitter), which are generally accessible by the public. The link to the survey was also circulated through emails and WhatsApp groups. Reminders and reposting of the links on social media were done on a regular basis. A letter describing the objectives of the study and assuring the confidentiality and anonymity of the collected data was attached to the survey. After potential participants read the letter attached to the survey, agreement to participate was sought by asking respondents to click a button. Clicking the button then took the participants to the survey questions. Ethical approval was obtained from the Institutional Review Board (IRB) of Hamad Medical Corporation.

### Study Questionnaire

The questionnaire was adapted from other validated and reliable questionnaires.^17,18^ It was initially developed in English and then translated into 3 other languages (Arabic, Malayalam, and Urdu) by an accredited translator. Face and content validities of the questionnaire were assured by experts in the field. The questionnaire consisted of 3 sections. The first section explored the sociodemographic characteristics of the participants (age, sex, nationality, marital status, highest level of education, employment status, and whether they worked from home or not during home confinement, and the presence of chronic diseases). Other sections explored substance use, including status and changes in tobacco and alcohol use and associated underlying factors, and changes in sleep duration, sleep quality, and social connectedness since the start of the restrictive measures.

### Outcome Measures

We focused on measuring the perceived changes in tobacco use and factors associated with such changes by asking participants to report whether they perceived an overall increase, decrease, or no change in the amount of tobacco used, to report the average amount used before and during the restrictive measures by selecting an amount category (<10, 10 to 20, 21 to 40, and >40 cigarettes/day) for cigarettes, and (≤ 1, 2 to 3, 4 to 6, and ≥7 times/week) for shisha (molasses-sweetened tobacco smoked in a hookah pipe), and to report the underlying causes for such changes in use. Participants also reported their overall perception of alcohol intake (increased, decreased, or remained the same). Sleep duration was assessed by asking participants to report the average hours of sleep per day before and during the restrictive measures. We assessed sleep quality by asking participants to rate their overall subjective sleep quality on a 5-point Likert scale: 1 (very good), 2 (good), 3 (average), 4 (poor), and 5 (very poor). Participants were also asked to indicate their degree of agreement with 6 statements concerning sleep latency, disturbances, and daytime dysfunction using a 4-point Likert scale: 1 (strongly disagree), 2 (disagree), 3 (agree), and 4 (strongly agree). A total score was calculated by summing the scores on the 6 statements, with higher scores indicating poorer sleep quality with a maximum score of 24. Social connectedness during restrictive measures was assessed using a similar agreement scale using a different set of statements. These statements were: “I feel disconnected from the world around me,” “I feel so distant from people,” and “I catch myself losing all sense of connectedness with society.”

### Statistical Analysis

Data analysis was performed using the Statistical Product and Service Solutions (SPSS) Version 26.0 (IBM Corp, Armonk, NY). Descriptive statistics were presented as frequencies and percentages for categorical variables. The χ^2^ test was used to assess differences in the overall perceived changes in tobacco and alcohol use between different groups. After testing for normality using the Shapiro-Wilk test, we used the nonparametric Wilcoxon Signed Rank test to detect differences in the amount of tobacco use, sleep duration, and subjective sleep quality before and during the restrictive measures. Rank biserial correlation was calculated to measure the effect size for these comparisons (small 0.10 to <0.30, medium 0.30 to <0.50, and large ≥0.50). The Mann-Whitney *U* test and the Kruskal-Wallis test were applied to compare the scores for sleep quality and social connectedness between groups, as the scores were not normally distributed. Multivariable logistic regression analysis was conducted to explore predictors of adverse changes in outcome variables. The Hosmer-Lemeshow test was used to assess the goodness of fit of the model. *P*-values <0.05 were considered statistically significant.

## RESULTS

### Sociodemographic Characteristics

As shown in Table 1, the survey was completed by 1408 participants. Over half (825, 58.6%) of the questionnaires were completed in English, followed by 23.2%, 17.3%, and 0.9% in Malayalam, Arabic, and Urdu, respectively. Males accounted for 58.8% of the total sample, with a male-to-female ratio of 1.4:1. The majority of participants (n=1084, 77%) were 25 to 44 years old, 1132 (80.4%) were married, and 1107 (78.6%) had completed a college degree or higher. Over 50 nationalities were reported, with the top 3 being Indians (53.4%), Filipino (5.2%), and Qatari (4.3%). Of the total participants, 1070 (76%) were employed, with 53.1% having shifted to work from home during the restrictive measures. About one-fifth (306, 21.7%) had a history of one or more chronic diseases.

**TABLE 1:** Sociodemographic Profiles and Background Information of the Participants (N=1408)

Variables	N (%)
Age, y	
18-24	57 (4.0)
25-34	540 (38.4)
35-44	544 (38.6)
45-54	203 (14.4)
55-64	59 (4.2)
65+	5 (0.4)
Sex	
Male	828 (58.8)
Female	580 (41.2)
Nationality (classification by regions)^*^	
Americas	45 (3.2)
Sub-Saharan Africa	54 (3.8)
Europe	117 (8.3)
Middle East—North Africa	273 (19.4)
Asia—Pacific	919 (65.3)
Highest level of education	
No formal education	18 (1.3)
High school diploma	228 (16.2)
College or higher	1107 (78.6)
Vocational training	55 (3.9)
Marital status	
Married	1132 (80.4)
Not married	276 (19.6)
Presence of chronic disease/s^†^	
Yes	306 (21.7)
No	1102 (78.3)
Employment related information	
Employment status	
Employed	1070 (76.0)
Not employed	338 (24.0)
Nature of work^‡^	
Mostly office work	746 (69.7)
Mostly field work	324 (30.3)
Working from home as part of “staying at home” measures^‡^	
Yes	568 (53.1)
No	502 (46.9)

* More than 50 different nationalities were reported.

† Most commonly reported chronic diseases were: diabetes, hypertension, asthma, and cardiovascular diseases.

‡ Denominator is the number of employed participants (n=1070).

### Substance Use During Restrictive Measures

Of the 1408 participants, 200 (14.2%) reported regular use of tobacco products (including smokeless tobacco). Of these, 72 (36%) perceived an increase in tobacco use, 53 (26.5%) perceived a decrease, and the remaining perceived no change in their use since the start of the restrictive measures. In assessing the change in the amount of tobacco used before and during the restrictive measures, about half (48.6%) of the 72 who perceived an increase in their use, reported a significant increase in the number of cigarettes (jumped into a higher amount category compared with before the restrictive measures), with *P*=0.035 but with a small effect size (*r*=0.28). While 22.2% reported an increase in shisha use (jumped into a higher amount category), the reported increase in shisha use was not significant *P*=0.843. On the other hand, among those who reported decreasing their smoking, one-third (32.1%) reported decreasing their cigarette amount to a lower category, and 22.6% reported decreasing their shisha use. For the remaining participants who perceived an increase or a decrease in their tobacco use, the change was either within the same amount category or in the use of other tobacco products. We detected a significant difference in reports of increased use between males and females (*P*=0.008), between those who perceived more stress during restrictive measures and those who did not (*P*=0.002), and between different nationalities (*P*=0.002). However, multivariable logistic regression showed that perceived stress was the only predictor for increased tobacco use during the restrictive measures [adjusted odds ratio (OR): 2.88, 95% CI: 1.32-6.31, *P*=0.008] (Table 2). Participants attributed the change in tobacco use to different factors. Many related their increase in use to feelings of boredom during restrictive measures (73.6%), worries about their employment and financial status during the pandemic (62.5%), and increased arguments with other family members during the restrictive measures (50%). Few reported stress about the current pandemic, homeschooling of children, and separation from family as triggers for their increase in tobacco use. Among those who decreased their tobacco use, 45.3% attributed this change to decreased social gatherings and less time spent with friends who smoke, 43.4% to fear of catching COVID-19 infection as smoking increases the risk, and 32.1% to pressure by family members to quit smoking. Few related their decrease in tobacco use to limited accessibility to tobacco products, and limited ability to buy such products in light of the financial consequences of COVID-19.

**TABLE 2:** Differences in the Use of Tobacco Products During COVID-19-related Home Confinement Measures Among Different Sociodemographic Subgroups (N=200)

	Tobacco use during home confinement measures				
				Multivariable logistic regression	
Variable	No increase, N (%)	Increase, N (%)	*P* ^*^	AOR^†^ (95%CI)	*P*
Age, y					
18-24	1 (33.3)	2 (66.7)	0.770	1 [reference]	
25-34	53 (64.6)	29 (35.4)		0.24 (0.01-3.97)	0.318
35-44	52 (65.0)	28 (35.0)		0.23 (0.1-3.88)	0.309
45-54	18 (66.7)	9 (33.3)		0.19 (0.01-3.63)	0.269
55+^‡^	4 (50.0)	4 (50.0)		0.43 (0.02-9.67)	0.597
Sex					
Male	109 (68.6)	50 (31.4)	**0.008**	0.62 (0.25-1.58)	0.320
Female	19 (46.3)	22 (53.7)		1 [reference]	
Nationality (classification by regions)					
Americas	3 (30.0)	7 (70.0)	**0.002**	5.03 (0.98-25.78)	0.053
Sub-Saharan Africa	4 (57.1)	3 (42.9)		1.88 (0.33-1.68)	0.475
Europe	15 (55.6)	12 (44.4)		2.09 (0.68-6.43)	0.200
Middle East—North Africa	32 (53.3)	28 (46.7)		2.16 (0.99-4.70)	0.051
Asia—Pacific	74 (77.1)	22 (22.9)		1 [reference]	
Highest level of education					
No formal education	1 (50.0)	1 (50.0)	0.428	0.54 (0.22-13.04)	0.703
High school diploma	25 (67.6)	12 (32.4)		0.31 (0.63-1.55)	0.154
College or higher	98 (64.9)	53 (35.1)		0.30 (0.07-1.27)	0.102
Vocational training	4 (40.0)	6 (60.0)		1 [reference]	
Employment status during confinement measures					
Employed					
Switched to working from home	52 (65.8)	27 (34.2)	0.139	0.82 (0.30-2.27)	0.706
Continued working regularly	61 (67.8)	29 (32.2)		0.82 (0.28-2.40)	0.711
Not employed	15 (48.4)	16 (51.6)		1 [reference]	
Marital status					
Married	102 (65.4)	54 (34.6)	0.442	0.97 (0.43-2.17)	0.934
Not married	26 (59.1)	18 (40.9)		1 [reference]	
Presence of chronic disease/s					
Yes	30 (68.2)	14 (31.8)	0.513	0.94 (0.40-2.22)	0.890
No	98 (62.8)	58 (37.2)		1 [reference]	
Perceived stress					
Yes	80 (57.1)	60 (42.9)	**0.002**	2.88 (1.32-6.31)	**0.008**
No	48 (80.0)	12 (20.0)		1 [reference]	

*P* values in bold indicate statistically significant results.

* Using χ^2^ test or Fisher exact test.

† Adjusted for all variables in the table.

‡ We combined 55 to 64 and 65+ categories into one category 55+, because of the very low number of smokers in the 65+ age group.

AOR indicates adjusted odds ratio.

Of the 1408 participants, 255 (18.1%) reported regular use of alcohol. Of these, 105 (41.2%) reported decreased alcohol use during the restrictive measures, while 14.5% reported an increase. Males were significantly more likely to report a decrease in their alcohol use compared with females (*P=*0.022). Multivariable logistic regression analysis showed that sex, nationality, and marital status were predictors of decreased use of alcohol during the restrictive measures (Table 3).

**TABLE 3:** Differences in the Use of Alcohol During COVID-19-related Home Confinement Measures Among Different Sociodemographic Subgroups (N=255)

	Alcohol use during home confinement measures				
				Multivariable logistic regression	
Variables	No decrease, N (%)	Decrease, N (%)	*P* ^*^	AOR^†^ (95% CI)	*P*
Age					
18-24	1 (33.3)	2 (66.7)	0.223	1 [reference]	
25-34	36 (53.7)	31 (46.3)		1.22 (0.25-5.98)	0.803
35-44	63 (55.3)	51 (44.7)		2.04 (0.4-10.15)	0.383
45-54	36 (70.6)	15 (29.4)		1.20 (0.22-6.56)	0.831
55+^‡^	14 (70.0)	6 (30.0)		1.81 (0.30-11.11)	0.521
Sex					
Male	86 (53.4)	75 (46.6)	**0.022**	1.95 (1.15-3.30)	**0.013**
Female	64 (68.1)	30 (31.9)		1 [reference]	
Nationality (classification by regions)					
Americas	15 (68.2)	7 (31.8)	0.102	2.61 (1.04-6.55)	**0.040**
Sub-Saharan Africa	4 (50.0)	4 (50.0)		1.05 (0.35-3.10)	0.933
Europe	45 (71.4)	18 (28.6)		2.50 (1.32-4.74)	**0.005**
Middle East—North Africa	9 (60.0)	6 (40.0)		0.27 (0.11-0.63)	**0.003**
Asia—Pacific	77 (52.4)	70 (47.6)		1 [reference]	
Highest degree of education					
No formal education	2 (66.7)	1 (33.3)	0.708	0.76 (0.8-7.15)	0.809
High school diploma	11 (47.8)	12 (52.2)		0.64 (0.21-1.92)	0.423
College or higher	131 (60.1)	87 (39.9)		1.012 (0.39-2.66)	0.981
Vocational training	6 (54.5)	5 (45.5)		1 [reference]	
Employment status during confinement measures					
Employed					
Switched to working from home	77 (60.2)	51 (39.8)	**0.036**	1.57 (0.76-3.22)	0.220
Continued working regularly	41 (49.4)	42 (50.6)		1.65 (0.77-3.53)	0.198
Not employed	32 (72.7)	12 (27.3)		1 [reference]	
Marital status					
Married	123 (61.2)	78 (38.8)	0.138	0.52 (0.31-0.89)	**0.016**
Not married	27 (50.0)	27 (50.0)		1 [reference]	
Presence of chronic disease/s					
Yes	35 (54.7)	29 (45.3)	0.437	1.54 (0.95-2.52)	0.082
No	115 (60.2)	76 (39.8)		1 [reference]	
Perceived stress					
Yes	100 (61.0)	64 (39.0)	0.349	1.20 (0.79-1.84)	0.394
No	50 (54.9)	41 (45.1)		1 [reference]	

*P* values in bold indicate statistically significant results.

* Using χ^2^ test or Fisher exact test.

† Adjusted for all variables in the table.

‡ We combined 55 to 64 and 65+ categories into one category 55+, because of the very low number of alcohol users in the 65+ age group.

AOR indicates adjusted odds ratio.

### Sleep and Social Connectedness During Restrictive Measures

Of the 1408 participants, 649 (46.1%) reported an increase in average sleep duration per day since the start of the restrictive measures and 218 (15.5%) reported a decrease. The mean sleep duration per day significantly increased from 6.95 hours/day before to 7.72 hours/day during the restrictive measures (mean increase of 0.77 h, 95% CI: 0.66-0.88, *P*<0.001) with a large effect size (*r*=0.54). With regard to overall subjective sleep quality, 416 (29.5%) reported a significant decrease in sleep quality since the start of the restrictive measures (*P*<0.001) with a small effect size (*r*=0.21). Disturbance in the sleep-wake cycle, with more daytime than nighttime sleep, was reported by 38.1% of the participants. Indeed, 18.9% even reported daytime dysfunction and having trouble staying awake while driving, eating meals, or engaging in social activities. Participants also reported an increase in sleep onset latency, with difficulty falling asleep (44.3%). Group analysis with pairwise comparisons showed higher percentages of participants with poorer sleep quality (higher sleep quality scores) among those aged 18 to 34 years of age compared with those 35 to 55 years of age, among nationalities of Middle Eastern-North African origin compared with those of Asia-Pacific origin, and among those not employed, unmarried, and those experiencing more stress during the pandemic compared with the others. The last 2 categories were also more likely to include higher percentages of participants with poorer social connectedness scores (Table 4). The majority (62.9%) stated that they felt distant from people since the start of the restrictive measures, 53.3% felt disconnected from the world around them, and 49.4% felt that they were losing their sense of connectedness with society. Over one-third (39.6%) agreed or strongly agreed with these 3 statements.

**TABLE 4:** Differences in Sleep Quality and Social Connectedness Scores Among Different Sociodemographic Subgroups During COVID-19-related Home Confinement Measures

Variables	Sleep quality score median (Q1-Q3)	*P*	Social connectedness score median (Q1-Q3)	*P*
Age, y				
18-24	16 (13-18)	**0.009**	8 (6-10)	**<0.001**
25-34	14 (12-17)		8 (6-9)	
35-44	14 (12-16)		8 (6-9)	
45-54	14 (12-16)		8 (6-9)	
55-64	13 (11-15)		7 (6-9)	
65+	13 (13-17)		9 (8-10)	
Sex				
Male	14 (12-16)	0.097	8 (6-9)	0.096
Female	14 (12-17)		8 (6-9)	
Nationality (classification by regions)				
Americas	13 (12-17)	**0.031**	8 (7-9)	0.224
Sub-Saharan Africa	14 (12-17)		8 (6-9)	
Europe	14 (12-16)		8 (6-9)	
Middle East—North Africa	15 (12-18)		8 (6-10)	
Asia—Pacific	14 (12-16)		8 (6-9)	
Highest level of education				
No formal education	14.5 (13-18)	0.744	7 (6-9)	0.211
High school diploma	14 (12-16)		8 (6-9)	
Vocational training	14 (12-17)		8 (6-9)	
College or higher	14 (12-17)		8 (6-9)	
Employment status during “staying at home” measures				
Employed				
Switched to working from home	14 (12-16)	**0.022**	8 (6-9)	0.058
Continued working regularly	14 (12-16)		8 (6-9)	
Not employed	14 (12-17)		8 (6-10)	
Marital status				
Married	14 (12-16)	**<0.001**	8 (6-9)	**0.014**
Unmarried	15 (12.5-18)		8 (6-10)	
Presence of chronic disease/s				
Yes	14 (12-17)	0.16	8 (6-9)	0.789
No	14 (12-16)		8 (6-9)	
Perceived stress				
Yes	15 (13-17)	**<0.001**	9 (7-10)	**<0.001**
No	13 (12-15)		6 (6-8)	

*P* values in bold indicate statistically significant results.

Q1 indicates quartile 1 (25th percentile); Q3, quartile 3 (75th percentile).

Multivariate logistic regression showed that participants who continued to work regularly during restrictive measures were less likely to report a worsening in sleep quality than those who switched to working from home (adjusted OR: 0.68, 95% CI: 0.46-0.99, *P=*0.045). Those who perceived more stress during the pandemic and home confinement were 4 times more likely to report worsening in their sleep quality than those who did not (adjusted OR: 4.04, 95% CI: 3.07-5.31, *P*<0.001). Participants in higher age groups and those who continued working regularly were less likely to report increased sleep duration compared with those 18 to 24 years of age and those who switched to work from home, respectively. Finally, participants who reported perceived stress were more likely to report increased sleep duration compared with those who did not (adjusted OR: 1.32, 95% CI: 1.05-1.64, *P*=0.016).

## DISCUSSION

The strict measures applied by countries around the globe such as restrictions on movement and other restrictive measures were intended to contain the spread of COVID-19 and keep people safe from getting infected. However, such measures could have indirect adverse impacts on several aspects of people’s lifestyles. In this population-based study, we tried to understand the triple “S” impact of COVID-19-related restrictive measures (on substance use including tobacco and alcohol use, sleep quality and duration, and social connectedness) among the general public of Qatar. The prevalence of smoking among Qatar’s population in our study was 14.2% which matches the most recently reported figure.^19^ Of the smokers, 36% reported an increase in tobacco use since the start of the restrictive measures, reinforcing the results of earlier studies that reported an increase in smoking during periods of lockdown.^20–22^ Persistence of this finding (ie, continued higher rate of tobacco use) in the post-pandemic era could result in an increase or worsening of smoking-related non-communicable diseases. In this study, perceived stress during the COVID-19 pandemic was found to be an independent predictor of increased tobacco use during periods of restrictive measures, confirming existing evidence that highlights stress as a risk factor for smoking.^12,13,23^ Encouraging healthy coping strategies to deal with stressors during such an unprecedented crisis is highly recommended. A large proportion of participants reported a decrease in alcohol drinking during restrictive measures. This may be explained by limited physical and financial accessibility to alcohol during the pandemic and the closure of clubs and bars in Qatar in an attempt to contain the spread of the infection.

A significant increase in average sleep duration per day was reported by participants during restrictive measures with a 0.77 hour mean increase, which is higher than what was reported in a study conducted among several European countries that showed a 0.25 hour mean increase in sleep duration during lockdown.^24^ However, the same study showed a reduction in sleep quality among one-third of participants which was similar to our findings.^24^ Other studies have also demonstrated a deterioration in sleep quality during periods of COVID-19 lockdown.^25,26^ We found disturbances in the sleep-wake cycle and prolonged sleep onset latency among participants, similar to findings in studies conducted in India that showed delayed sleep onset, a reduction in nighttime sleep duration, and increased daytime napping.^26,27^ Evolving evidence suggests a bidirectional association between sleep and infection.^28,29^ Sleep deprivation and poor sleep quality adversely affect the immune response to infectious agents. Therefore, maintaining adequate and optimal sleep quality may be critical to protecting against the SARS-CoV-2 virus. Another bidirectional relationship exists between sleep and stress.^30^ Unsurprisingly, we found in our study that perceived stress was a predictor of worsening sleep quality among the participants. This finding may be explained by the adverse impact of the pandemic on sleep and circadian rhythms. However, healthy sleep was critically needed to cope adaptively with this crisis. Participants who continued working regularly and did not switch to working from home were less likely to perceive a worsening in their subjective sleep quality than those who did. This could probably be attributed to the disruption of usual routines among those who switched to working from home, which could have adversely affected their sleep. Having to deal with homeschooling of kids, especially with the closure of schools during the pandemic, the blurred and diminished work-life boundaries for those working from home, and not having a limit for working hours can contribute to a build-up of stress, which can further impact sleep quality and push people toward unhealthy behaviors including substance use. Optimum sleep quality is critically important for recovering from stressful life events and we believe that it is needed to cope well in the postpandemic era, as evidence suggests that poor sleep is associated with prolonged recovery from a stressful event.^31^

Social connectedness is a critical aspect of lifestyle that was adversely affected during the restrictive measures as evidenced by this study. Increased use of social media during periods of lockdown as a way of communication while movement was restricted has been noted worldwide.^32,33^ However, despite being one of the few means of communication in the context of strict restrictive measures, use of social media had a deleterious effect on the overall social well-being of individuals, induced undue stress among communities, and adversely impacted life satisfaction.^32,34^ This might indicate that connecting with people virtually through social media platforms cannot replace direct face-to-face social interactions. We initially expected to find a significant difference in social connectedness scores between Qataris and non-Qataris, since the majority of non-Qataris are far away from their homes, families, and friends. However, such a difference could not be demonstrated in this study, possibly because of the small percentage of Qataris (4.3%) in our sample compared with other nationalities.

Some similarities in the sociodemographic characteristics of our participants can increase the ability to generalize from the findings of this study. We found that about 91% of the participants were between 25 and 54 years of age, which is similar to the age distribution in the general population, where 93.7% of Qatar’s population above 24 years of age are between 25 and 54 years old, while 4.7% are between 55 and 64 years old.^35,36^ Moreover, non-Arabs comprise over 70% of the population in Qatar, which is consistent with the findings in our study in which over 80% of the participants were non-Arabs.^37^ The fact that over three-quarters of the sample reported being highly educated (with a college degree or higher) makes us more confident in generalizing the results to educated people.

It is evident that the SARS-CoV-2 virus can mutate in different ways that can result in accelerated virus transmission and reduced vaccine effectiveness. This could seriously result in repeated outbreak recurrences given the continuous appearance of new and challenging SARS-CoV-2 variants and existing vaccine hesitancy.^38^ The history of previous infectious outbreaks and pandemics has proved that COVID-19 will not be the last pandemic, particularly with continued population growth, climate change, globalization, and international travel that serve as triggers for emerging infections.^39^ With the possibility that movement restrictions may be reimplemented, whether during new waves of the COVID-19 pandemic or during any other potential infectious outbreak or emergency, countries should implement health promotion and lifestyle interventions to minimize substance use during such stressful times.

The strict measures taken to contain the spread of COVID-19 could have serious indirect repercussions on different aspects of people’s lifestyles. Further research is needed to address whether such negative consequences will persist or decline in the postpandemic era.

### Strengths and Limitations

This study had several strengths. As a national study, we were able to reach an acceptable sample size of 1408 participants. This study was one of the few conducted in the Middle East to address substance use, sleep, and social connectedness (the 3 “Ss”) in the context of the COVID-19-related restrictive measures. In addition, data were collected in 4 languages which can be considered a point of strength in a multilingual country like Qatar.

However, we also acknowledge several limitations. First, the sampling technique we used might have introduced selection bias compromising the representativeness of the sample. The link to the survey was posted on the social medial platforms of Hamad Medical Corporation. It is possible that people who follow the social media pages of health institutions are more cautious about their health and more likely to adopt healthy behaviors, which might bias the results. However, during the pandemic, Hamad Medical Corporation was the main source of credible information about the COVID-19 pandemic besides the Ministry of Public Health and provided daily updates about the pandemic and about restrictive and preventive measures. We therefore believe that, during the pandemic, many people regardless of their health behaviors were interested in receiving updates about COVID-19 and were following HMC social media platforms, and this might have somewhat minimized the possibility of bias related to the health behaviors of participants. Second, the data obtained were self-reported and liable to information bias and social desirability bias. Social desirability bias might have underestimated the adverse lifestyle behaviors. Having to compare the amount of tobacco use, sleep duration, and sleep quality before and after the implementation of the restrictive measures might also have resulted in recall bias. However, using a web-based, self-administered questionnaire was the only way to explore this area of research given the physical and social distancing measures recommended during the pandemic.

## CONCLUSIONS

This study illustrated the adverse impact of COVID-19-related restrictive measures on important aspects of the lifestyle of Qatar’s population with regard to substance use, sleep, and social connectedness. Over one-third of tobacco users perceived an increase in tobacco use since the start of the restrictive measures, and almost half of those reported a significant increase in the amount of cigarette smoking. This finding, if the increased use continued, could lead to an increase in or worsening of smoking-related noncommunicable diseases. Fortunately, over 40% of alcohol users reported decreased alcohol use. One-third reported a deterioration in subjective sleep quality, and about 40% felt that they were socially disconnected. We found that perceived stress was an independent risk factor for increased tobacco use and worsening of subjective sleep quality. Although restrictive measures were critically important to contain the spread of COVID-19 infection, evidence derived from this study should be taken into consideration in developing future regulations in Qatar. More emphasis should be given to encouraging people to maintain healthy lifestyle behaviors during restrictive measures that may be imposed during any public health crises or any potential future infectious outbreaks through raising public awareness. Public awareness messages should focus on avoiding unhealthy behaviors such as tobacco and alcohol use, maintaining sleep hygiene to diminish the impact on sleep quality, and adopting healthy coping strategies to deal with various stressors during such challenging times. Researchers need to focus now on addressing the persistence of such adverse consequences in the postpandemic era. Evidence from this study should also further stimulate efforts directed toward the implementation of effective lifestyle-related interventions.
